# The role of wearable technologies in supporting physical and psychosocial health outcomes among breast cancer patients: a systematic review

**DOI:** 10.1007/s00520-026-10554-9

**Published:** 2026-03-14

**Authors:** Tuğba Şahin Tokatlıoğlu, Arzu Kavala, Fahriye Oflaz

**Affiliations:** 1 Faculty of Health Sciences, Department of Nursing, Istanbul Aydın University, Istanbul, Turkey; 2 School of Nursing, Koc University, Istanbul, Turkey

**Keywords:** Breast cancer, Wearable Technology, Physical Activity, Psychological Adaptation, Cancer Survivors

## Abstract

**Purpose:**

This systematic review aimed to examine the effects of wearable technologies on physical functioning, symptom-related outcomes, and psychosocial health parameters in patients with breast cancer. The primary research question was whether wearable devices provide added value as supportive tools when integrated with established interventions such as exercise or mindfulness-based approaches.

**Methods:**

The review was registered in the PROSPERO database (CRD420251113029). A systematic search was conducted across Cochrane Library, PubMed, Web of Science, Scopus, and Ovid MEDLINE. Randomized controlled trials evaluating wearable technologies in breast cancer populations were included. Study selection and data extraction were independently conducted by two reviewers using the Covidence platform. Due to heterogeneity in interventions and outcomes, results were synthesized using a narrative approach.

**Results:**

Three randomized controlled trials published between 2019 and 2024 met the inclusion criteria, with sample sizes ranging from 28 to 52 participants. Wearable technologies included EEG headbands, activity trackers, and smart bracelets. Interventions supported by wearable devices were associated with improvements in fatigue, emotional and functional domains of quality of life, perceived stress, and physical activity. In exercise-based studies, objectively measured moderate-to-vigorous physical activity increased (e.g., median O-MVPA: 234.3 vs. 128.3 min/week), and cardiorespiratory fitness improved (VO₂peak + 2.43 mL/kg/min). High feasibility and user acceptability were consistently reported.

**Conclusion:**

Findings from a limited number of randomized controlled trials suggest that wearable technologies may play a supportive role in breast cancer care by facilitating monitoring, adherence, and self-management when combined with established interventions. However, the available evidence remains limited, and further high-quality research is needed to clarify the independent and additive contributions of wearable technologies to physical, clinical, and psychosocial outcomes.

**Supplementary Information:**

The online version contains supplementary material available at 10.1007/s00520-026-10554-9.

## Background

Breast cancer is not only one of the most commonly diagnosed and challenging malignancies worldwide but also represents a significant public health concern [1]. It has surpassed lung cancer to become the most prevalent malignant tumor globally [2]. Although advancements in early-stage detection and metastatic disease have improved outcomes, substantial challenges related to treatment side effects persist [3, 4]. Surgical procedures and the removal of breast tissue often lead to negative body image perceptions among patients [5], while adjuvant therapies may result in fatigue, reduced physical capacity, increased pain, lymphedema, hair loss, gastrointestinal symptoms, and menopausal complaints. Given the impact of these symptoms on treatment adherence, quality of life, body image, and morbidity and mortality rates, effective symptom management remains a critical component of cancer care [6, 7]. The primary goal of symptom management in breast cancer patients is to prevent or delay adverse physical, psychological, and social outcomes. Systematic identification, assessment, and control of patients’ symptoms are critical for delivering consistent and effective care [8]. In particular, cancer patients should be supported to actively participate in daily life activities and manage symptoms and treatment-related side effects at home [9]. In recent years, pioneering research has been conducted in cancer care technology to address these needs and develop innovative solutions [10]. Technological approaches used in cancer symptom management include various medical devices, software, mobile applications, and wearable technologies. These technologies can assist patients in coping with the physical, emotional, and cognitive challenges they encounter during treatment [11].

One of these technologies is wearable technology. Wearable technologies provide patients and healthcare professionals involved in their care with personalized and real-time health data [12]. Wearable devices can provide clinicians with objective information on a patient’s health status prior to treatment, enable early detection of adverse events, and facilitate monitoring of adherence to prehabilitation and rehabilitation programs. The rich data collected by these devices can assist healthcare professionals in monitoring individuals’ health status, including sleep quality, healthy posture, cognitive decline, and even early warning signs of infection and inflammation. In the field of oncology, wearable devices can contribute to the improvement of cancer care management by providing new and critical information on patients’ health status, such as heart rate, blood pressure, activity level, sleep quality, and behavioral activities [13–15]. Although not originally designed for medical purposes, such devices have the potential to support remote monitoring, require minimal user compliance, and offer objective health information [16]. In a scoping review examining the use of wearable technologies in patients with breast cancer, adherence rates to wearable devices were reported to range from 60 to 100%. In addition, among 10 studies that assessed patient-reported outcomes using questionnaires and individual interviews, 8 demonstrated a positive association between patient-reported outcomes and data from wearable devices [17]. Wearable technologies have predominantly been utilized in individuals diagnosed with breast cancer for early detection [18], monitoring fatigue [16], and tracking physical activity [19, 20]. These technologies are considered potentially beneficial for breast cancer patients due to their ability to offer more frequent monitoring, particularly in individuals at moderate to high risk, and to facilitate earlier identification of abnormalities and timely interventions [14, 21]. Recent studies using microwave-based wearable sensors have demonstrated their potential for early breast cancer detection at the technical and diagnostic levels, successfully differentiating malignant from healthy tissue based on dielectric properties and achieving promising classification accuracy. However, the impact of these technologies on long-term clinical outcomes has not yet been established. In this context, given the high cost of cancer treatment, the use of wearable devices, including for early detection, may also be cost-effective, as they enable personalized and flexible treatment plans [21–23].

Wearable technologies enable the continuous and objective monitoring of symptoms and daily activities, which may allow for earlier identification of symptom deterioration and support patients’ engagement in self-management outside clinical settings. By providing real-time feedback and longitudinal data, these devices may enhance symptom awareness, strengthen monitoring processes, and facilitate communication with healthcare professionals, thereby indirectly supporting treatment adherence. However, despite growing interest in wearable technologies in cancer care, evidence regarding how and to what extent these technologies influence treatment adherence, symptom management, and psychosocial outcomes remains limited and fragmented. Existing studies vary substantially in their intervention designs, outcome measures, and reported adherence, underscoring the need for a systematic synthesis of the available evidence to better understand the role of wearable technologies in breast cancer care.

## Methods

### Study design

This review adhered to the PRISMA (Preferred Reporting Items for Systematic Reviews and Meta-Analyses) guidelines. The protocol was registered with PROSPERO (CRD420251113029), and the entire research process was conducted in accordance with the methods outlined in the protocol. This systematic review was conducted in accordance with the Joanna Briggs Institute (JBI) guidelines for systematic reviews [24, 25]. The review was guided by a focused research question developed using the PICO framework:

What evidence exists regarding the use of wearable technologies for symptom management and psychosocial outcomes in patients with breast cancer?

This question informed the development of the search strategy, inclusion criteria, and data extraction procedures. The search strategy will be described separately. Owing to heterogeneity in study designs, outcome measures, and sample sizes, a meta-analysis was not conducted. As pre-specified in the review protocol, a narrative (descriptive) synthesis was performed when quantitative synthesis was not feasible. This synthesis was informed by key principles of the SWiM (Synthesis Without Meta-analysis) reporting guideline, with findings grouped according to intervention characteristics, outcome domains (symptom-related and psychosocial outcomes), and feasibility-related measures.

### Needs assessment and topic selection

Despite growing interest in the use of wearable technologies in cancer care, particularly for symptom monitoring, uncertainty remains regarding how and to what extent these technologies influence symptom management, treatment adherence, and psychosocial outcomes in women with breast cancer. Symptom burden and fluctuations during and after treatment may pose challenges to sustained self-management and adherence, especially in long-term and home-based care settings.

Wearable technologies may offer potential support in this context by enabling continuous, objective monitoring of symptoms and daily activities, facilitating earlier identification of symptom deterioration, and supporting patient engagement outside clinical environments. Through real-time feedback and longitudinal data collection, wearables may indirectly support symptom awareness, monitoring, and communication with healthcare professionals. However, the mechanisms through which these potential benefits translate into improved adherence or psychosocial adjustment remain insufficiently understood.

Existing studies in this field are heterogeneous in terms of intervention design, outcome measures, and reported adherence, and often focus on isolated outcomes, small sample sizes, or short-term follow-up periods. Consequently, there is a lack of comprehensive synthesis of the available evidence that systematically examines both physical and psychosocial outcomes in breast cancer populations. These gaps highlight the need for a methodologically rigorous systematic review to critically evaluate the current evidence, clarify what is known and unknown about the role of wearable technologies in breast cancer care, and identify directions for future research.

### Study development

This systematic review was conducted using a structured seven-step methodological framework to ensure transparency and scientific rigor. The process included: (1) identifying the research need and selecting the topic, (2) developing the study protocol, (3) performing a comprehensive literature search, (4) screening and selecting eligible studies, (5) extracting relevant data, and (6) assessing the risk of bias. The entire review process was conducted in strict accordance with the PRISMA guidelines [24, 25] to enhance methodological quality.

### Literature search

**Fig. 1:**
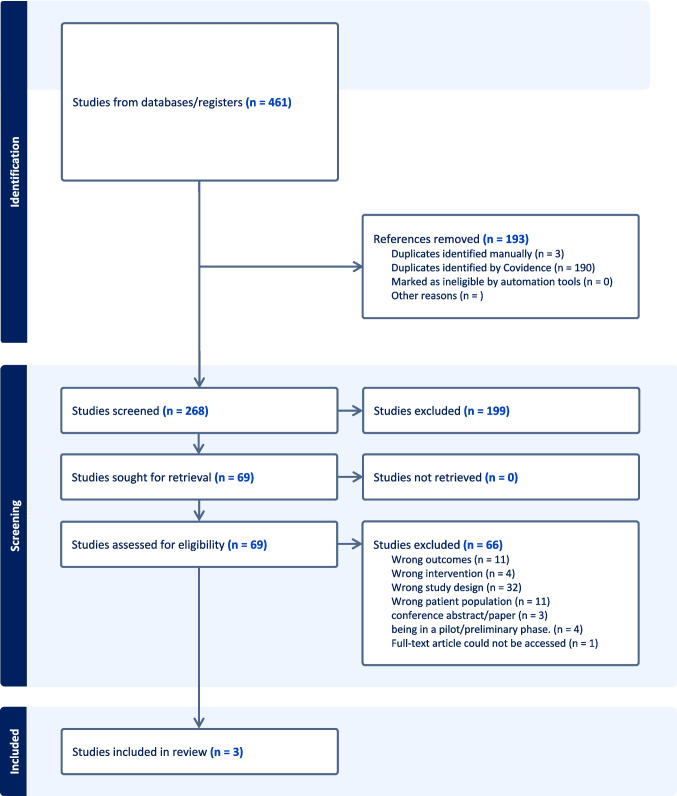
PRISMA

This systematic review followed the Preferred Reporting Items for Systematic Reviews and Meta-Analyses (PRISMA) guidelines [24] (Fig. 1) to ensure a comprehensive and systematic approach to identifying relevant studies. A structured literature search was conducted across multiple electronic databases, including PubMed, Cochrane Library, Web of Science, Scopus, and Ovid MEDLINE, to identify peer-reviewed studies evaluating the impact of wearable technologies on symptom management and psychosocial adjustment in patients with breast cancer.

The search strategy was developed using Medical Subject Headings (MeSH) terms and relevant keywords to capture studies evaluating the impact of wearable technologies on symptom management and psychosocial adjustment in breast cancer patients. Boolean operators (AND, OR) were used to combine terms effectively. The main search terms were structured as follows:

#1: “Breast Neoplasms”[Mesh] OR “breast cancer” OR “breast tumor” OR “cancer of breast”.

AND #2: “Wearable Electronic Devices”[Mesh] OR “sensor technolog*” OR “wearable technology” OR “remote health monitoring” OR “wearable device”.

AND #3: “Adjustment Disorders”[Mesh] OR “psychosocial adjustment” OR “reactive disorders” OR “reactive depression*”Searches were conducted across the following electronic databases: PubMed, Cochrane Library, Web of Science, Scopus, and Ovid MEDLINE. The search was limited to studies published in English.

After pilot searches, the third search block (#3) did not yield any additional eligible records when combined with the other search terms using the AND operator (0 additional results). It was retained in the search strategy to ensure conceptual completeness and transparency. The complete, expanded search strategies applied across all databases, along with the corresponding search results, are presented in Appendix 1.

In addition to database searches, a manual review of the reference lists of relevant systematic reviews and included studies was conducted to identify additional eligible articles. Grey literature (e.g., conference abstracts and institutional reports) was not included due to feasibility constraints.

### Study selection and inclusion criteria

#### Eligibility criteria

The inclusion and exclusion criteria for this systematic review were defined using the PICOS framework (Population, Intervention, Comparison, Outcome, Study Design) [26]:

Population: Patients diagnosed with breast cancer

Intervention: Use of wearable technologies

Comparison: Standard care or other conventional interventions

Outcomes: Psychosocial outcomes, symptom-related outcomes, and physical functioning parameters associated with wearable technology use in breast cancer patients

Study Design: Randomized controlled trials (RCTs)

### Screening and data extraction

Two reviewers (AK and TŞT) independently conducted the title and abstract screening and full-text assessment using the Covidence platform. Covidence provides three decision options during screening: “Yes,” “No,” and “Maybe.” When one reviewer selected “Yes” and the other “No,” the system flagged the record as a conflict requiring resolution prior to progression. Conflicts were resolved through discussion between the reviewers. A third reviewer (FO) was included in discussions regarding any conflicts or ambiguities in the results during the data extraction process.

During the full-text screening stage, both reviewers again made independent judgments regarding eligibility. If discrepancies arose in exclusion reasons, Covidence flagged them for joint resolution. These conflict management mechanisms supported consistency, transparency, and alignment with systematic review methodology (see Table 1).

**Table 1:** Distribution of included studies by characteristics (*n* = 3)

Title of the study	Author name-year-country	Aim of the study	Sample	Inclusion criteria	Study design	Applied wearable technology device	Follow-up duration	Assessment tools	Findings, conclusion
1. Use of a Wearable EEG Headband as a Meditation Device for Women With Newly Diagnosed Breast Cancer: A Randomized Controlled Trial [27]	Millstine, 2019 USA	This study aims to use a portable, wearable, electroencephalographic device for guided meditation practices by breast cancer patients during the period from breast cancer diagnosis until 3 months after surgical treatment	28 patients	Women aged 20–75 years with newly diagnosed breast cancer, scheduled for surgical treatment, and able to complete ≥ 7 days of intervention prior to the presurgical visit	Randomized controlled trial	Muse mindfulness headband	Baseline, pre-surgery, post-surgery (≤ 14 days), and 3 months post-surgery	Multidimensional Fatigue Symptom Inventory–Short Form (MFSI-SF) Functional Assessment of Cancer Therapy–General (FACT-G) Perceived Stress Scale (PSS) Was It Worth It (WIWI) questionnaire	Participants in the intervention group showed significant within-group improvements in fatigue (MFSI-SF total score: 20.5 to 3.6), perceived stress (PSS: 16.0 to 10.1), and emotional well-being (FACT-G emotional subscale: 17.3 to 20.9) over a 3-month follow-up period; however, between-group differences were not statistically significant
2. Feasibility and effect of a physical activity counselling session with or without provision of an activity tracker on maintenance of physical activity in women with breast cancer—A randomised controlled trial [28]	Singh, 2020 Australia	To evaluate the effect and feasibility of physical activity counselling with or without an activity tracker (Fitbit) on physical activity maintenance over 12 weeks in women with breast cancer following a supervised exercise intervention	52 patients	Women with stage II–IV breast cancer who had completed the SAFE supervised exercise trial, were physically inactive, resided in the greater Brisbane region, and were either undergoing treatment or had completed treatment within the previous 5 years	Randomized controlled trial	Activity tracker (Fitbit Charge HR®)	12 weeks	Active Australia Survey; ActiGraph GT3X + accelerometer; Fitbit use and acceptability questionnaire	Provision of a wearable activity tracker (Fitbit) following supervised exercise was feasible and acceptable, with high device use (> 80%) over a 12-week follow-up. Compared with counselling alone, the PAC + Fitbit group showed higher objectively measured MVPA at 12 weeks (median O-MVPA: 234.3 vs. 128.3 min/week, *p* = 0.03) and higher self-reported total physical activity (between-group difference at 12 weeks: 171.9 min/week [95% CI 46.1–297.8]; group × time *p* < 0.01)
3. Improving physical and mental health in women with breast cancer undergoing anthracycline-based chemotherapy through wearable device-based aerobic exercise: a randomized controlled trial [29]	Li, 2024 China	To evaluate the efficacy, safety, and adherence of moderate to high-intensity aerobic exercise facilitated by wearable devices on physical and mental health outcomes in women with breast cancer undergoing anthracycline-based chemotherapy	41 patients (planned), 40 completed	Female patients aged 30–65 years with stage I–III breast cancer receiving anthracycline-based chemotherapy at Sanhuan Cancer Hospital	Randomized controlled trial	Smart bracelet (Huawei) integrated with the MicroMotion Manager mobile application, used to monitor heart rate, guide exercise intensity, record exercise sessions, assess adherence, and enable remote supervision during chemotherapy	12 weeks (across four cycles of anthracycline-based chemotherapy)	Body composition assessed using bioelectrical impedance analysis (InBody 770) Cardiorespiratory fitness (VO₂peak) assessed by cardiopulmonary exercise testing (CPET) Handgrip strength measured using an electronic handgrip dynamometer Physical activity energy expenditure assessed using the International Physical Activity Questionnaire–Short Form (IPAQ-SF) Anxiety and depression assessed using the Self-Rating Anxiety Scale (SAS) and Self-Rating Depression Scale (SDS) Cancer-related fatigue assessed using the Cancer Fatigue Scale (CFS) Sleep quality assessed using the Pittsburgh Sleep Quality Index (PSQI) Quality of life assessed using the Chinese version of the Functional Assessment of Cancer Therapy–Breast (FACT-B, V4.0) Exercise adherence and intensity monitored using a wearable smart bracelet (heart rate and session data)	Wearable device–guided moderate to high-intensity aerobic exercise performed three times per week for 12 weeks during anthracycline-based chemotherapy was found to be safe, feasible, and effective. Compared with usual care, the exercise group demonstrated significantly greater improvements in cardiorespiratory fitness (VO₂peak + 2.43 mL/kg/min, *p* = 0.003) and handgrip strength (+ 7.10 kg, *p* < 0.001), as well as significant reductions in anxiety (− 5.74, *p* = 0.007), depression (− 7.00, *p* = 0.028), cancer-related fatigue, and sleep disturbances. Quality of life also improved, particularly in functional and emotional well-being domains. Exercise adherence was high (mean adherence 81.8%), no serious exercise-related adverse events were observed, and the wearable device enabled real-time monitoring of exercise intensity and adherence, supporting safe implementation during chemotherapy

Covidence was also used to streamline the workflow, manage citations, and ensure precise documentation throughout the review process. The PRISMA flow diagram (Fig. 1) outlines the number of records identified, screened, and excluded, and the reasons for exclusion.

### Risk of bias assessment

The methodological quality of the included studies was independently assessed by two authors (AK and TŞT) using the Joanna Briggs Institute Meta-Analysis of Statistics Assessment and Review Instrument (JBI–MAStARI). In randomized controlled trials (RCTs), data quality was evaluated using a scoring system that assigned 1 point per applicable item, with a maximum possible score of 13. Each item was rated as “Yes,” “No,” “Unclear,” or “Not Applicable.” The overall risk of bias for each study was independently determined by the two reviewers. Any discrepancies were resolved through discussion and consensus (Table 2). Due to heterogeneity in study designs, interventions, and outcome measures, a meta-analysis was not conducted. Instead, a narrative synthesis was performed to summarize the findings across studies.

**Table 2:** Risk of bias assessment of the studies

**Joanna Briggs Institute Critical Appraisal Checklist for Randomized Controlled Trial Studies**															
**First author (year)**	**Q1**	**Q2**	**Q3**	**Q4**	**Q5**	**Q6**	**Q7**	**Q8**	**Q9**	**Q10**	**Q11**	**Q12**	**Q13**	**Final quality score**	**Rating**
Denise M. Millstine, 2019	Y	Y	Y	Y	Y	Y	Y	Y	Y	Y	Y	Y	Y	13	100%
Ben Singh, 2020	Y	Y	Y	Y	U	U	U	Y	Y	Y	Y	Y	Y	10	76.9%
Hongmei Li, 2024	Y	Y	Y	Y	N	Y	Y	Y	Y	Y	Y	Y	Y	12	92.3%

## Results

### Search results and study selection

A total of 461 records were identified through database searches using predefined keywords. After duplicate records were removed, 268 records remained. These were initially screened by the reviewers based on title and abstract, using an independent, blinded process in which the reviewers were unaware of each other’s decisions. As a result of this phase, 199 records were excluded by consensus.

The remaining 69 full-text articles were independently assessed by both reviewers, again using a blinded review platform. Following full-text evaluation, 3 studies met the inclusion criteria and were included in the final systematic review (Table 1).

### Characteristics of studies and participants

All three studies included in the systematic review involved women undergoing treatment for breast cancer. In the study by Millstine [27], the mean age of participants was 55.9 years (SD = 11.1), with 60% reporting a history of depressive symptoms. Regarding surgical intervention, 53.3% of the participants had undergone lumpectomy, while 46.7% had received a mastectomy. In the study conducted by Singh [28], the mean age of the 52 participants was 52.8 years (SD = 9.5). The majority of participants (84.7%–92.4%) had completed active treatment, and 65.4% had undergone mastectomy. Treatment history revealed that 88.5%–92.3% had received chemotherapy, 80.7%–88.5% had undergone radiotherapy, and 73.1%–80.8% had received hormone therapy, either previously or currently. The median time since diagnosis was 20 months (range: 5–82 months), and disease stages were primarily stage II (46.2%) and stage III (34.6%–38.4%). In the study by Li [29], the mean participant age was 48.47 years, with tumor staging distributed as follows: 5.3% stage I, 42.1% stage II, and 52.6% stage III. These data indicate that the studies included in this review generally involved heterogeneous groups of patients, most of whom were diagnosed with advanced-stage breast cancer and had undergone or were undergoing various treatment modalities.

### Characteristics of intervention

The included studies were conducted between 2019 and 2024, with sample sizes ranging from 28 to 52 participants. One study was conducted in the USA [27], one in Australia [28], and one in China [29]. All three studies were randomized controlled trials. The wearable technologies used in these studies included a wearable EEG device, a smart bracelet, and physical activity trackers (Fitbit).

More comprehensive information about the interventions implemented in the studies is presented in Table 1.

### Summary of included studies

Three randomized controlled trials were included in this systematic review, evaluating wearable technology–supported interventions in women with breast cancer across different clinical settings.

Millstine et al. [27] evaluated the use of a wearable EEG headband (Muse) for guided meditation from diagnosis to three months after surgery. Outcomes were assessed at baseline, pre-surgery, post-surgery, and 3 months post-surgery using standardized instruments for fatigue (MFSI-SF), quality of life (FACT-G), and perceived stress (PSS). The intervention group demonstrated within-group reductions in total fatigue scores (MFSI-SF: 20.5 to 3.6) and perceived stress (PSS: 16.0 to 10.1), along with improvements in emotional well-being over the follow-up period; however, between-group differences were not statistically significant.

Singh et al. [28] examined the effect of physical activity counselling with or without a wearable activity tracker (Fitbit) over a 12-week follow-up period. Compared with counselling alone, the counselling plus Fitbit group demonstrated higher objectively measured moderate-to-vigorous physical activity at 12 weeks (median O-MVPA: 234.3 vs. 128.3 min/week, *p* = 0.03) and higher self-reported total physical activity (between-group difference: 171.9 min/week, 95% CI 46.1–297.8; *p* < 0.01). A greater proportion of participants in the Fitbit group met national physical activity recommendations at follow-up (77% vs. 52% for total activity). Device feasibility was high, with over 80% of participants reporting regular device use throughout the intervention period.

Li et al. [29] investigated a wearable device–guided moderate to high-intensity aerobic exercise intervention during anthracycline-based chemotherapy over 12 weeks. Compared with the control group, the exercise group showed significantly greater improvements in cardiorespiratory fitness (VO₂peak + 2.43 mL/kg/min, *p* = 0.003) and handgrip strength (+ 7.10 kg, *p* < 0.001), as well as significant reductions in anxiety (− 5.74, *p* = 0.007), depression (− 7.00, *p* = 0.028), cancer-related fatigue, and sleep disturbances. Exercise adherence was high (mean adherence 81.8%), and no serious exercise-related adverse events were reported.

### Quality assessment results

All three randomized controlled trials included in this review demonstrated good methodological quality, as indicated by their quality assessment scores. A detailed summary of the assessment is provided in Table 2.

## Discussion

In this systematic review, we examined the effects of wearable technologies such as EEG devices, smart bracelets, and physical activity trackers (e.g., Fitbit) on physical functioning, symptom-related outcomes (e.g., fatigue, sleep disturbances), and psychosocial outcomes (e.g., stress, anxiety, depression, and quality of life) in patients with breast cancer. Importantly, in the included studies, wearable technologies were primarily used as monitoring and adherence-enhancing tools alongside established interventions, rather than as standalone therapeutic modalities. While breast cancer patients maintain their health through primary treatments such as surgery and chemotherapy, they often face challenges during and after treatment, including fatigue, sleep disturbances, stress, depression, fear, and decreased self-esteem [29–31]. These challenges can significantly affect patients’ adaptation processes and quality of life, potentially hindering their recovery [32]. Therefore, wearable technologies aim to improve patients’ quality of life by providing various benefits ranging from early detection and symptom management to encouraging active participation in health maintenance [33].

Fatigue is one of the most prominent symptoms associated with breast cancer and its treatment, and it negatively affects patients’ quality of life [34].The National Comprehensive Cancer Network defines fatigue as “a distressing, persistent, subjective sense of physical, emotional, and/or cognitive tiredness or exhaustion related to cancer or cancer treatment that is not proportional to recent activity and interferes with usual functioning” [35].

Although rest is often considered sufficient for coping with fatigue, a lack of physical activity may increase the risk of muscle weakness and functional decline [36]. Increasing physical activity has been shown to reduce perceived fatigue [37]. Physical activity is considered the first-line intervention for cancer-related fatigue and has been found to be more effective than pharmacological or psychological treatments [38, 39].

In a study examining physical activity maintenance among women with breast cancer, the addition of a wearable activity tracker to counselling was associated with higher objectively measured and self-reported physical activity at 12-week follow-up compared with counselling alone, suggesting a supportive role of the device in monitoring and adherence rather than a direct therapeutic effect [28]. In the randomized controlled study by Li et al. [29], moderate to high-intensity aerobic exercise performed during chemotherapy was shown to result in significant improvements in cardiorespiratory fitness, handgrip strength, and physical activity levels compared with the control group, along with corresponding reductions in anxiety, depression, cancer-related fatigue, and sleep disturbance scores. In this study, the wearable device was used primarily as a supportive tool to monitor exercise intensity and adherence rather than as the primary driver of the intervention’s effects. Similarly, in the study conducted by Millstine et al. [27] which utilized a wearable EEG headband as a meditation device, fatigue levels were found to be reduced at two weeks and three months post-surgery compared to baseline. These findings suggest that wearable technologies are feasible and may support the effective implementation of exercise or mindfulness-based interventions. The associated increase in physical activity appears to contribute to reductions in symptoms such as fatigue, anxiety, depression, and insomnia, potentially contributing to improvements in symptom burden and psychosocial well-being.

Sleep disturbances are a common symptom experienced by breast cancer patients during and after chemotherapy [40]. Numerous factors can trigger or exacerbate cancer-related insomnia, including stress resulting from the diagnosis and treatment process, side effects of medications and chemotherapy, reduced physical activity, and disruption of circadian rhythms [41, 42]. The presence of sleep problems not only increases the risk of psychiatric and physical comorbidities but also diminishes patients’ motivation to complete treatment, thereby reducing their overall quality of life [43]. In the study conducted by Li et al. [29] breast cancer patients monitored through wearable device-based aerobic exercise interventions demonstrated decreased sleep disturbance scores and improved sleep quality. These findings suggest that wearable device-guided aerobic exercise during chemotherapy may serve as a supportive adjunct to enhance adherence and facilitate improvements in physical and mental health outcomes. Anxiety and depression are highly prevalent among patients with breast cancer and are associated with poorer quality of life [29]. In the study by Millstine et al. [27], use of a wearable EEG headband as a meditation aid was associated with reductions in stress and improvements in quality of life at two weeks and three months postoperatively compared with baseline. Similarly, Li et al. [29] reported that patients in the exercise group demonstrated significantly lower anxiety and depression scores compared with the control group following the intervention. Importantly, these improvements were attributed to the aerobic exercise intervention itself, while the wearable device primarily facilitated monitoring of exercise intensity and adherence rather than acting as an independent therapeutic modality.

Taken together, these findings suggest that wearable technologies may play a supportive role in the management of psychological symptoms in breast cancer patients by enabling adherence to evidence-based interventions such as exercise or mindfulness, rather than directly exerting therapeutic effects on anxiety or depression. Gastrointestinal reactions such as loss of appetite, nausea and vomiting, difficulty in eating, diarrhea, and oral ulcers are commonly observed in breast cancer patients undergoing treatment [29, 44]. These symptoms negatively impact patients’ nutritional status, physical and mental health, and most notably, their quality of life [28]. In the study by Li et al. [29], the frequency and severity of gastrointestinal reactions were reported to be lower in the group that performed aerobic exercise during chemotherapy compared with the control group. However, this effect is attributable to the exercise intervention itself, while the role of the wearable device was primarily limited to monitoring exercise intensity and adherence. These findings suggest that aerobic exercise performed during chemotherapy may be associated with reductions in gastrointestinal symptoms, while wearable technologies primarily supported monitoring and adherence. Breast cancer patients require comprehensive and holistic care to maintain their quality of life, including both physical and social dimensions [45, 46]. The World Health Organization (WHO) emphasizes the importance of quality of life in breast cancer care, particularly with respect to emotional, social, and role functioning [47]. In this context, wearable technologies have been proposed as tools that may support the monitoring of key patient-reported outcomes, such as physical activity, sleep quality, fatigue, and stress-related symptoms during treatment [29].

Evidence from the limited number of available studies suggests that the use of wearable devices alongside established interventions (e.g., exercise or mindfulness-based approaches) may be associated with improvements in quality-of-life outcomes among breast cancer patients [27–29]. However, these benefits appear to be primarily driven by the underlying interventions, with wearable technologies serving a supportive role by facilitating monitoring, adherence, and self-management rather than acting as independent therapeutic agents.

Taken together, these findings highlight the potential supportive value of wearable technologies in breast cancer care, while also underscoring the need for further high-quality research to clarify their specific contribution to quality-of-life outcomes. The inclusion of only three studies in the present systematic review indicates that the evidence regarding the effects of wearable technologies in breast cancer care remains limited. A review of the broader literature suggests that much of the existing research in this field is still at the pilot or prototype development and feasibility stage [16, 21]. This highlights an ongoing gap between the proposed potential of wearable technologies and the availability of large-scale, high-quality randomized controlled trials capable of demonstrating their effectiveness, clinical relevance, and specific contributions across diverse clinical populations.

Beyond efficacy considerations, the integration of continuous monitoring and IoT-based data collection into clinical practice offers opportunities to enhance decision-making but simultaneously raises challenges related to data burden, false-positive alerts, patient acceptance, privacy, and ethical governance [48, 48–50]. Accordingly, the integration of wearable technologies into routine breast cancer care should be approached not only as a technological innovation but also as a socio-technical process that requires alignment with healthcare delivery models, ethical standards, and data governance frameworks.

### Strengths and limitations

This review possesses several notable strengths. To the best of our knowledge, it is the first systematic review to evaluate the effects of wearable technologies on symptom management and psychosocial adjustment in breast cancer patients. The study’s methodological robustness is enhanced by the use of a pre-registered protocol, a comprehensive and systematic literature search, and the application of established quality appraisal tools. Furthermore, by including a wide range of clinical and psychosocial outcomes—such as fatigue, sleep quality, anxiety, depression, gastrointestinal symptoms, and physical functioning—this review provides a broad and integrated perspective on how wearable technologies contribute to improving patient well-being. The inclusion of various wearable devices (e.g., activity trackers, EEG headbands, smartwatches) further enriches the generalizability and relevance of the findings across different technological applications.

However, several limitations should be acknowledged. This systematic review included only randomized controlled trials conducted with breast cancer patients. However, the literature search revealed a limited number of such studies in this area, and many were still in the pilot phase. As a result, only three studies met the inclusion criteria for this review.

Restricting inclusion to randomized controlled trials, while enhancing internal validity, may have led to the exclusion of relevant prospective, feasibility, or observational studies that reflect early-stage or real-world applications of wearable technologies. This represents a deliberate trade-off between methodological rigor and breadth of evidence and should be considered when interpreting the findings.

In addition, although a range of outcomes was examined, these outcomes were not uniformly assessed across studies, and the observed effects were largely attributable to the underlying interventions (e.g., exercise or meditation), with wearable technologies primarily serving a supportive role in monitoring, adherence, and self-management rather than acting as independent therapeutic agents.

Additionally, only studies published in English were included, which may have introduced language bias and led to the exclusion of potentially relevant findings from non-English sources.

Finally, although the reviewers were blinded to each other’s decisions within the Covidence platform, they were not blinded to study authorship, journal names, or institutional affiliations, which could have introduced a potential source of bias.

## Conclusion

This systematic review suggests that wearable technology devices may be useful as supportive tools in managing symptoms, enhancing quality of life, and strengthening psychosocial adjustment among patients with breast cancer. The studies reviewed indicate potential benefits of these technologies have particularly positive effects in monitoring and alleviating treatment-related challenges such as fatigue, sleep disturbances, anxiety, depression, and gastrointestinal symptoms, particularly when used alongside established interventions such as exercise or mindfulness-based approaches.

Additionally, wearable technologies may support patients’ engagement in health-related behaviors, including physical activity, thereby facilitating self-monitoring and self-management rather than directly producing therapeutic effects. Continuous monitoring and feedback mechanisms provided through wearable devices appear to assist with adherence to prescribed interventions and symptom tracking, which may contribute to psychosocial well-being.

From a clinical perspective, wearable technologies have the potential to support nurses and healthcare professionals by enabling more continuous symptom monitoring and data-informed assessments. However, current evidence does not yet allow firm conclusions regarding their role in guiding clinical decision-making or early interventions, and their use should be considered complementary to standard care.

Importantly, the heterogeneity of study protocols, small sample sizes, and the limited number of eligible studies restrict the strength and generalizability of the available evidence. Therefore, conclusions regarding effectiveness should be interpreted with caution. Future research should prioritize well-designed, larger-scale randomized controlled trials that clearly distinguish the effects of the underlying interventions from the supportive role of wearable technologies, as well as examine long-term outcomes, cost-effectiveness, and feasibility in routine clinical practice. At present, wearable technologies may be considered promising adjunctive tools rather than established components of breast cancer care.

## Supplementary Information

Below is the link to the electronic supplementary material.

ESM 1 (DOCX 14.8 KB)

## Data Availability

No datasets were generated or analyzed during the current study.
