# Supplementary material for: The role of wearable technologies in supporting physical and psychosocial health outcomes among breast cancer patients: a systematic review
Source: Support Care Cancer. 2026 Mar 14;34(4):324. doi: 10.1007/s00520-026-10554-9 (PMC12988973; doi:10.1007/s00520-026-10554-9)
Supplement: Supplementary file 1 — (DOCX 14.8 KB) [file 520_2026_10554_MOESM1_ESM.docx]

**Pubmed**

3 ("Breast Neoplasms"[Mesh] OR "breast cancer" OR "Breast tumor" OR "Cancer of Breast") AND ("Wearable Electronic Devices"[Mesh] OR "Sensor technolog*" OR "wearable technology" OR "remote health monitoring" OR "wearable device") ("Breast Neoplasms"[MeSH Terms] OR "breast cancer"[All Fields] OR "Breast tumor"[All Fields] OR "Cancer of Breast"[All Fields]) AND ("Wearable Electronic Devices"[MeSH Terms] OR "sensor technolog*"[All Fields] OR "wearable technology"[All Fields] OR "remote health monitoring"[All Fields] OR "wearable device"[All Fields]) 99

2 "Wearable Electronic Devices"[Mesh] OR "Sensor technolog*" OR "wearable technology" OR "remote health monitoring" OR "wearable device" "Wearable Electronic Devices"[MeSH Terms] OR "sensor technolog*"[All Fields] OR "wearable technology"[All Fields] OR "remote health monitoring"[All Fields] OR "wearable device"[All Fields] 29,333

1 "Breast Neoplasms"[Mesh] OR "breast cancer" OR "Breast tumor" OR "Cancer of Breast" "Breast Neoplasms"[MeSH Terms] OR "breast cancer"[All Fields] OR "Breast tumor"[All Fields] OR "Cancer of Breast"[All Fields] 487,743

**Cochrane Library**

#1 "breast cancer" OR "Breast tumor" OR "Cancer of Breast" 45557

#2 Sensor NEXT technolog* OR "wearable technology" OR "remote health monitoring" OR "wearable device" 948

#3 #1 AND #2 25

**Web of Science**

1: TS=("breast cancer" OR "Breast tumor" OR "Cancer of Breast") Date Run: Mon Feb 24 2025 13:31:06 GMT+0300 (GMT+03:00) Results: 660378

2: TS=("Sensor technolog*" OR "wearable technology" OR "remote health monitoring" OR "wearable device") Date Run: Mon Feb 24 2025 13:31:16 GMT+0300 (GMT+03:00) Results: 25560

3: #2 AND #1 Date Run: Mon Feb 24 2025 13:31:24 GMT+0300 (GMT+03:00) Results: 101

**Scopus**

TITLE-ABS-KEY ( "breast cancer" OR "Breast tumor" OR "Cancer of Breast" ) AND TITLE-ABS-KEY ( "Sensor technolog*" OR "wearable technology" OR "remote health monitoring" OR "wearable device" )

168

**Ovid MEDLINE(R) and Epub Ahead of Print, In-Process, In-Data-Review & Other Non-Indexed Citations, Daily and Versions <1946 to February 20, 2025>**

1 breast cancer.mp. or Breast Neoplasms/ 478419

2 Breast tumor.mp. 13026

3 Cancer of Breast.mp. 9990

4 1 or 2 or 3 480946

5 Sensor technology.mp. 2023

6 wearable technology.mp. or Wearable Electronic Devices/ 11983

7 remote health monitoring.mp. 245

8 wearable device.mp. 2993

9 5 or 6 or 7 or 8 15758

10 4 and 9 68

**Total Records: 461**

**After Delete of Duplicate Records: 268**
